# Engineering 3D-printed standalone conductive nerve guides using soft bioinks for peripheral nerve injuries

**DOI:** 10.1039/d5ma00932d

**Published:** 2025-11-12

**Authors:** Lin Li, Angel Hernandez, Ryan Grevsmuehl, Yu-Ting Kou, Shang Song

**Affiliations:** a Department of Biomedical Engineering, College of Engineering, University of Arizona Tucson Arizona USA shangsong@arizona.edu; b Department of Materials Science and Engineering, Neuroscience GIDP, and BIO5 Institute, University of Arizona Tucson Arizona 85721 USA

## Abstract

Conductive nerve guides (CNGs) demonstrate significant regenerative capabilities for bridging critically sized nerve defects due to their unique electrical and mechanical characteristics. However, nerve guides fabricated from conducting polymers through conventional electrochemical methods present challenges, including non-biodegradability and limited customization potential. Here we demonstrate customizable 3D-printed CNGs fabricated using biocompatible bioinks composed of poly(3,4-ethylenedioxythiophene):polystyrene sulfonate (PEDOT:PSS) and polyvinyl alcohol (PVA) without the need for sacrificial support. The synthesized soft bioinks composed of PEDOT:PSS/20% PVA showed enhanced conductivity, wettability, and shear-thinning behavior. By tailoring the polymer concentration and polymerization conditions, the standalone CNGs, fabricated using extrusion-based 3D printing, were custom-made to match the dimensions of critically sized nerve defects in rodent PNI models. The elimination of sacrificial layers during 3D printing avoids complex post-processing material removal and potential residue-related cytotoxicity. As a result, the 3D-printed CNGs demonstrated excellent biodegradability and biocompatibility. Optimizing soft bioink properties offers a simple manufacturing approach for producing 3D-printed biodegradable and biocompatible CNGs with customizable dimensions. Our findings address the critical need for advanced nerve guide designs tailored to treat peripheral nerve injuries of varying defect sizes.

## Abstract 2

Development of customizable and biodegradable 3D-printed conductive nerve guides for irreversible peripheral nerve defects.

## Graphical abstract

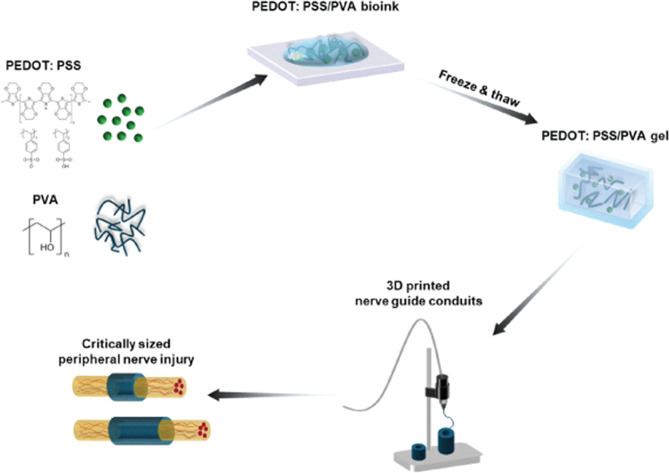

## 1 Introduction

Peripheral nerve injuries (PNIs) refer to damage to the peripheral nerves caused by motor vehicle accidents, combat trauma, and neurological disorders.^1,2^ Although the peripheral nervous system possesses a higher regenerative capacity than the central nervous system, functional recovery remains constrained due to potential neuroma formation, unguided axonal growth, and a limited regeneration rate of approximately 1–3 mm per day.^1,3^ Autologous nerve grafts are regarded as the gold standard for PNI treatment. The clinical application of this method is hindered by issues such as the scarcity of available nerve donors and donor site morbidity.^4^ Biomaterial-based nerve guide conduits (NGCs) have been extensively studied as alternatives to solve the above-mentioned constraints associated with autografts.^5,6^ NGCs made of natural components such as collagen and chitosan have shown ideal biodegradability and biocompatibility for peripheral nerve repair.^7^ However, their clinical applications are limited due to inadequate mechanical strength, potential immunogenic features, and suboptimal functional recovery outcomes.^8^

Electrical stimulation, particularly at low frequencies, has been shown to promote stem cell activity, guide cell migration, enhance neurite outgrowth, and support nerve regeneration and functional recovery.^9,10^ Furthermore, modulation of the electrical stimulation intensity and direction can influence cell orientation and migratory behavior.^11^ Conductive nerve guides (CNGs) that facilitate electrical transmission have demonstrated significant improvements in peripheral nerve regeneration and influenced long-term neurological functions.^12–14^ However, current conducting polymers are non-degradable, and the electrochemical synthesis of conducting polymers exhibits limited ability for customization.^13,15,16^ This is challenging in the context of PNI repair as the complexity and variability of nerve damage differ from person to person. Furthermore, commercial conduits are generally restricted to repairing short nerve gaps (*e.g.* below critically sized defects) and axonal dispersion remains a significant complication following transplantation.^17^ To address these challenges, researchers have been actively exploring innovative fabrication techniques to develop customizable CNGs for PNIs.

Three-dimensional (3D) printing enables the highest degree of freedom for design flexibility and offers a relatively cost-effective alternative for producing customizable scaffolds with greater reproducibility compared to other techniques.^18^ Sacrificial layers are frequently employed in 3D printing of hollow structures, as they reinforce the materials to prevent shifting or sliding during post-curing processing.^19^ These sacrificial layers can be utilized for 3D printing of complex shapes including single, bifurcated, irregular, or multi-lumen conduits.^20^ However, the presence of sacrificial layers requires extra post-processing to dissolve supporting molds, which can compromise the integrity of the main structure and introduce cytotoxic chemicals, potentially limiting the 3D printing application for clinical use.^21^

Here, we introduced a new perspective through the integration of extrusion-based 3D printing with a simple, single-component bioink formulation of poly(3,4-ethylenedioxythiophene):polystyrene sulfonate (PEDOT:PSS) and polyvinyl alcohol (PVA). PEDOT:PSS is recognized for its exceptional biocompatibility and electrochemical stability, making it one of the most widely used materials for neural interfaces.^14,22–24^ PVA is a hydrophilic biodegradable polymer that has been widely used in tissue engineering.^25^ PVA implants have been approved by the Food and Drug Administration (FDA) for clinical treatment, such as artificial metatarsophalangeal joint.^26,27^ By optimizing the polymer concentration and physical crosslinking of PEDOT:PSS/PVA bioinks, we successfully fabricated standalone 3D-printed CNGs with customizable heights and improved mechanical strength without sacrificial layers. Our bioink formulation and fabrication method reduced post-processing steps and complications associated with the cytotoxicity of sacrificial layer materials. Importantly, the standalone CNGs made of soft bioinks could be printed to match the critical dimensions used to treat critically sized nerve defects in rodent PNI models.

**Fig. 1:**
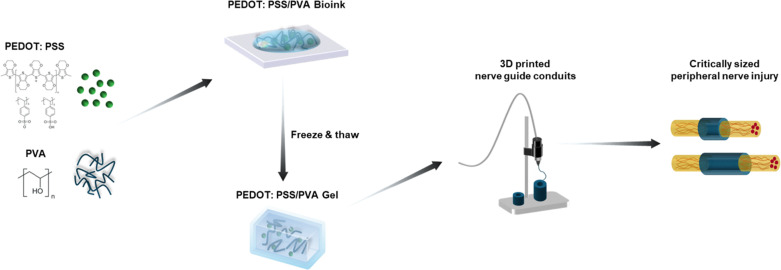
Schematic diagram of the fabrication process of 3D-printed nerve guide conduits and their potential application for peripheral nerve injury repair.

To evaluate the fabrication of 3D-printed standalone CNGs, we determined the microstructure and physical and mechanical properties of our synthesized bioinks. The combination of PEDOT:PSS and higher PVA concentration led to increased conductivity and viscosity of the bioinks. The PEDOT:PSS/20% PVA bioinks exhibited shear-thinning behavior favorable for extrusion-based 3D printing. Optimizing freeze–thaw cycle times further enhanced the physical crosslinking of the polymers and maintained the structural integrity of 3D-printed CNGs. The 3D-printed nerve guides demonstrated favorable biodegradability and biocompatibility, and the neural progenitor cells (NPCs) loaded within the 3D-printed CNGs showed enhanced neurotrophic factor secretion, which might further facilitate NPC proliferation and axonal growth.^28–31^ In summary, our results highlight the development of 3D-printed standalone CNGs composed of conductive and biodegradable bioinks, providing a simple, cost-effective fabrication strategy that maintains structural fidelity and bioactivity without requiring complex post-processing for treatments of various nerve defects in PNIs (Fig. 1).

## 2. Results and discussion

### 2.1. Fabrication and characterization of crosslinked bioinks

**Fig. 2:**
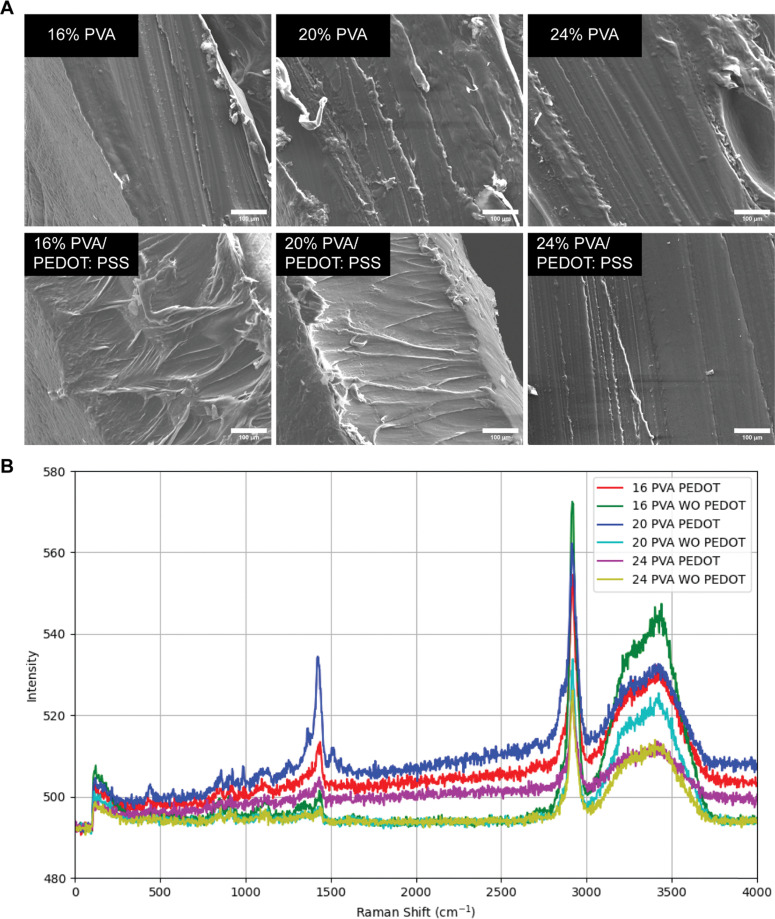
Characterization of bioinks. (A) SEM images of the cross-sectional surface of bioinks made of different concentrations of PVA (16%, 20%, and 24%) with or without PEDOT:PSS. (B) Raman spectroscopy analysis of bioinks made of different concentrations of PVA (16%, 20%, and 24%) with or without PEDOT:PSS. Scale bar = 100 µm.

The biodegradable bioinks were fabricated by mixing PEDOT:PSS with different concentrations of PVA (16%, 20%, and 24%). All the bioinks exhibited a smooth surface texture following crosslinking with no gross changes observed (Table S1). Scanning electron microscopy (SEM) was performed to examine the microstructure of the crosslinked bioinks. Results revealed that samples with higher concentrations of PVA showed more densely crosslinked internal structures, aligning with the previous publication.^32^ In the presence of PEDOT:PSS, the bioinks appeared similar to those without PEDOT:PSS, exhibiting similar smoothness and crosslinking densities (Fig. 2A). The presence of PEDOT:PSS had no impact on the crosslinked architecture of the bioinks, which may be attributed to the low concentration of PEDOT:PSS utilized in the system.

The molecular composition of the bioinks was detected by Raman spectroscopy (Fig. 2B). The spectra showed that lower PVA concentrations exhibited higher intensities at the 3400 cm^−1^ peak, which corresponds to O–H bonding.^33^ The reduced concentrations of PVA resulted in an effective increase in the relative water concentration in the collected samples, indicating that water balance exerted a greater impact on the molecular structure at reduced PVA densities. Therefore, changing the water content can potentially influence the physical properties of 3D-printed CNGs. Additionally, all sample groups exhibited high peaks at 2900 cm^−1^, representing C–H bonding, with a common trend of lower concentrations of PVA correlating with higher intensities.^34^ Samples with PEDOT:PSS showed peaks around 1450 cm^−1^, which were correlated with the presence of carbon–carbon double bonds (C=C), which were abundant in PEDOT:PSS thiophene rings.^35^ No significant vibration was observed among the six groups, indicating that the formation of the crosslinked bioinks was a physical process and that no new chemical bonds were generated.

Numerous nerve conduits have been developed over the past decades. Fang *et al.*^36^ reported a multiscale CNG with hierarchical fibers for nerve repair, and it exhibited a comparable outcome in neural functional recovery compared to autologous grafts in their animal models. PVA has seen an increase in use for 3D printing due to its ease of use, flowability, and cost-effectiveness.^37^ Recent attention has been directed toward developing novel PVA-based NGCs with enhanced properties. Jhang *et al.*^38^ reported an electrospun film made of PVA and carbon nanotubes, exhibiting favorable biocompatibility and conductivity for fabrication of NGCs. Stocco *et al.*^39^ developed a hybrid scaffold composed of oxidized PVA and bioactivated chitosan sponges, which was highly biocompatible in mouse models. Nevertheless, PVA itself lacks regenerative signals and exhibits limited ability for manipulation post-transplantation, and thus additional components are often required for fabrication of NGCs to achieve better therapeutic outcomes.^40^ PEDOT:PSS has garnered increasing interest for developing CNGs and scaffold conduits, with reported applications in neural engineering. PEDOT:PSS/PVA composites have been applied as active radiation-sensing compounds.^24^ Furlani *et al.*^41^ fabricated an injectable hydrogel combining PEDOT:PSS with gelatin, which exhibited excellent biocompatibility with rat astrocytes. Liu *et al.*^42^ developed porous PLGA fibers coated with PEDOT:PSS, which promoted peripheral motor function recovery in rats. Lu *et al.*^43^ fabricated a PEDOT:PSS/PVA/poly(acrylic acid) (PAA) film to improve the optrode–neural tissue interface. Babaie *et al.*^44^ developed a PVA/PEDOT scaffold using electrospun fibers, which promoted neural differentiation after electrical stimulation. We developed mechanically adaptive bioelectronics using PEDOT:PSS films to interface with peripheral nerves, enabling long-term neuromodulation in growing rats.^14^ We further explored different crosslinking additives to enhance the electrical and mechanical properties of PEDOT:PSS.^24^ Leveraging the advantageous properties of PVA and PEDOT:PSS, we developed bioinks with excellent conductivity, hydrophilicity, and mechanical properties, enabling their use in 3D printing applications. The versatility of 3D printing allowed us to create customizable CNGs for peripheral nerve injury repair. The high conductivity of the 3D-printed CNGs is highly desirable as it plays an important role in promoting nerve regrowth,^13,45^ while their robust mechanical properties will be critical to withstanding the natural physiological forces following injury.^46,47^

### 2.2. Physical and mechanical properties of crosslinked bioinks

**Fig. 3:**
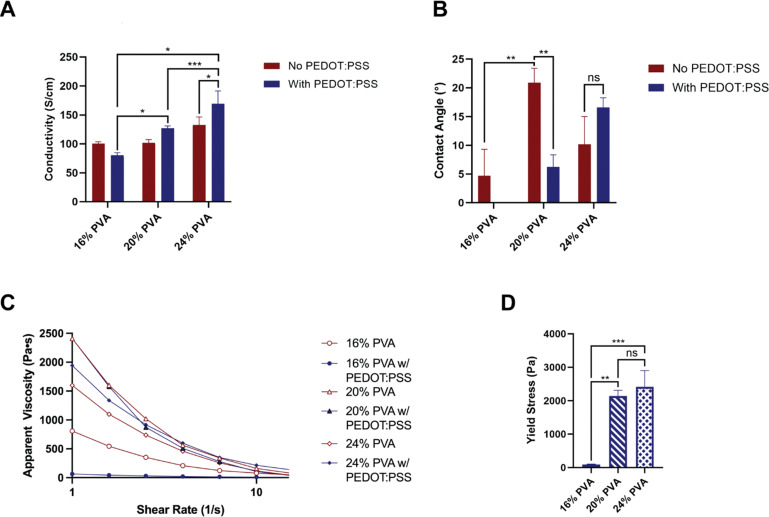
Physical and mechanical properties of the synthesized bioinks. (A) Conductivity, (B) contact angle, and (C) rheological characterization of bioinks composed of different concentrations of PVA (16%, 20%, and 24%) with or without PEDOT:PSS. (D) Mechanical properties of bioinks composed of different concentrations of PVA (16%, 20%, and 24%) with PEDOT:PSS. * *p* < 0.05, ** *p* < 0.01, and *** *p* < 0.001.

Conductivity measurements were performed on the formulated bioinks to characterize their inherent electrical properties. PVA concentration was identified as an independent factor influencing the conductivity of the bioink (*p* < 0.001), while PEDOT:PSS did not make a significant difference (*p* = 0.150). Although an increasing trend of conductivity was observed when PEDOT:PSS was added to the PVA bioinks, there was no statistically significant difference between the two paired groups. In the presence of PEDOT:PSS, 24% PVA bioinks showed significantly higher conductivity compared to those with 16% PVA (*p* < 0.001; Fig. 3A). The wettability of different bioinks was analyzed *via* contact angle measurement. PVA concentration showed independent effects (*p* = 0.002), while PEDOT:PSS had no particular effect on wettability (*p* = 0.106). However, for the 20% PVA formulation, the water contact angle was significantly reduced when PEDOT:PSS was introduced (*p* = 0.004). Upon the addition of PEDOT:PSS, bioinks with greater PVA concentrations showed elevated water contact angles, indicating increasing material hydrophobicity (Fig. 3B). The rheology of different bioinks was examined to evaluate viscosity and yield stress properties by oscillatory sweep tests. All bioinks displayed shear-thinning viscoelastic behavior demonstrated by the loss in viscosity with increasing applied shear rate following oscillatory rheological tests (Fig. 3C). Notably, the yield stress demonstrated a significant enhancement with increasing concentrations of PVA (*p* = 0.007; Fig. 3D).

The addition of PEDOT:PSS is known to increase electrical conductivity in polymer composites,^48,49^ whereas higher PVA content is known to increase crosslinking density.^32^ However, the small ratio of 0.1% PEDOT:PSS in PVA explained the insignificant increase in conductivity in this study. The concentration of PVA led to the development of bioinks exhibiting enhanced conductivity when combined with PEDOT:PSS. This might also be attributed to the increased crosslinked morphology and the more densely interconnected polymer networks.^50^

Biocompatibility is an essential requirement for any biomaterial intended for use in medical implants, and surface hydrophilicity is one of the key factors affecting the cell adhesion to polymers.^51,52^ When introducing PSS into PEDOT, PEDOT:PSS achieves better performance in supporting cell proliferation and adhesion.^23^ PVA is also considered as a highly hydrophilic polymer, and it has been applied as a surface coating to achieve a more hydrophilic surface.^53^ All bioinks demonstrated surface hydrophilicity in this study. Although PEDOT:PSS was not taken as an independent influential factor, the bioink composed of PEDOT:PSS and 20% PVA still showed a more hydrophilic characteristic compared to the 20% PVA bioink alone.

Given that the bioinks were predominantly composed of PVA, the elevation in PVA concentration substantially influenced the material properties due to the increased density of entangled polymer chains.^54^ This alteration also affected the conductivity, wettability, and mechanical properties of the bioinks.^32^ Although the bioink containing 24% PVA with PEDOT:PSS exhibited superior mechanical properties, its high viscosity may hinder printing viability due to the shear stress encountered within the nozzles.^55^ Consequently, the formulation of 20% PVA with PEDOT:PSS was deemed optimal for the 3D printing of CNGs, as it provided a favorable balance between conductivity, wettability, and the requisite shear-thinning viscoelastic behavior essential for extrusion-based 3D printing.

### 2.3. Mechanical properties and degradability of 3D-printed structures

**Fig. 4:**
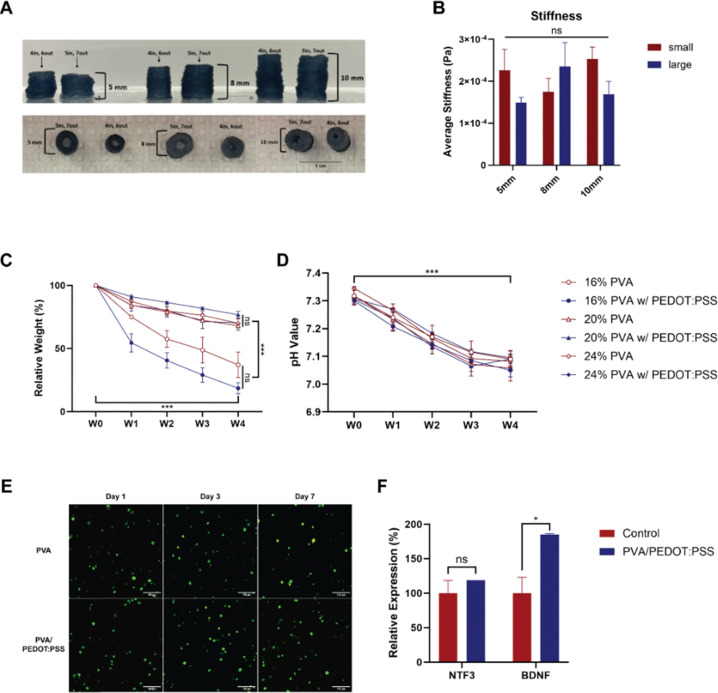
Mechanical properties and biodegradability of 3D-printed CNGs. (A) Side and top views of 3D-printed CNGs with different diameters and heights after freeze–thaw cycles. (B) Stiffness of different sizes of CNGs (small: internal diameter = 4 mm, external diameter = 6 mm; large: internal diameter = 5 mm, external diameter = 7 mm). (C) Relative weight loss and (D) changes in the pH value of CNGs composed of different concentrations of PVA with or without PEDOT:PSS in 4 weeks. (E) Cell live/dead staining on alginate-encapsulated human neural progenitor cells in 3D-printed CNGs composed of PVA and PVA/PEDOT:PSS. (F) qPCR results of neuronal markers NTF3 and BDNF of NPCs cultured in cell culture plates (control) and alginate-encapsulated in 3D-printed CNGs composed of PVA/PEDOT:PSS. * *p* < 0.05; scale bar = 100 µm.

A classical freeze–thaw method was employed as the primary mechanism for physical crosslinking of bioinks to achieve the structural integrity required for 3D-printed CNGs. The 3D extrusion-based printability of the 20% PVA with PEDOT:PSS bioink was evaluated by varying the freezing–thawing parameters. The influence of freezing time on the structural integrity of 3D-printed CNGs was assessed by direct visual comparison. A longer freezing time resulted in improved structural stability, enhanced printability, and denser crosslinking within the 3D-printed CNGs (Table S2). Hollow, cylindrical CNGs were printed without support materials and customized to match the dimensions needed to treat critically sized nerve defects in rodent PNI models (Fig. 4A). The 3D-printed standalone CNGs of varying dimensions were assessed for compressive stiffness. With a constant 2-mm width difference between the inner and outer diameters of the printed CNGs, minimal changes in material stiffness were observed when varying the CNG heights (*p* = 0.807) and sizes (*p* = 0.290; Fig. 4B). The challenge lay in the printing method used to construct standalone CNGs using soft polymer bioinks, which needed to be adjusted for their viscosity. To enhance the printability, we varied the freeze–thaw process by changing the freezing time of bioinks to enhance the underlying physical crosslinking.^56^ Increasing freezing time led to higher-crosslinked PVA, allowing for the fabrication of cylindrical CNGs with improved structural integrity. We further evaluated whether printed dimensions influenced the mechanical properties of 3D-printed CNGs. We modeled CNGs with dimensions matching the implantable nerve guides used to treat critically sized nerve defects in small animals.^13,14,57^ Compressive mechanical testing showed that the length and diameter of 3D-printed CNGs had no significant mechanical changes to printed structures.

The use of extrusion-based 3D bioprinting in our study offers several advantages over conventional methods that employ electrochemistry or electrospinning for nerve guide fabrication. Fabrication techniques, such as inkjet printing, screen printing, and electron-beam lithography, have been limited by various factors including low resolution, constrained two-dimensional patterning capabilities, and the intricate and costly nature of the processes.^49^ The 3D extrusion-based printing, demonstrated by our process, is more advantageous for nerve guide fabrication due to rapid prototyping, relatively inexpensive costs of materials, and high customization.^58–60^ Importantly, our CNGs can be fabricated with customizable dimensions without sacrificial layers, addressing the diverse needs for different peripheral nerve defects while reducing costs associated with post-processing steps in 3D printing.

To assess the degradability of the CNGs, changes in weight and pH levels were analyzed (Fig. 4C and D). Both the weight and pH values of the CNGs across all experimental groups exhibited a significant reduction after a four-week period (*p* < 0.001). In groups containing 20% and 24% PVA, with or without the addition of PEDOT:PSS, no significant differences in weight loss were observed. Conversely, CNGs formulated with 16% PVA exhibited greater weight loss compared to those with higher PVA concentrations (*p* < 0.001), suggesting that increased PVA concentrations contribute to enhanced stability. Cho *et al.* also reported that a soy protein isolate containing higher PVA concentration exhibited a longer degradation time because PVA took relatively longer time to degrade.^61^ The pH value showed a gradual decline from 7.3 to approximately 7.1 over the four-week duration, with no significant differences noted among the various groups. PEDOT:PSS is not classified as an officially biodegradable material; however, it can attain biodegradability by integrating biodegradable components, including silk fibroin, graphene, glycerol, and PVA.^62,63^ PVA possesses several advantageous features including high solubility and biodegradability, and thus it has been widely applied in the modification of various materials.^64^ Moreover, previous studies have shown that PVA exhibits excellent stability for at least three months in ophthalmic, orthopedic, and neural applications,^64–66^ which coincides with the critical recovery period of a 1 cm rat sciatic nerve defect,^67^ suggesting its suitability as a conduit material for guided nerve regeneration. Our findings indicated that the 3D-printed CNGs possess relative stability and could undergo gradual biodegradation, which would be helpful for further *in vivo* applications.

### 2.4. Biocompatibility of 3D-printed structures

Cellular components and growth factors play a crucial role in the process of neural regeneration following injury. Researchers have focused on enhancing the biological functionality of NGCs with cell supplementation emerging as a promising strategy. Several studies have reported that NGCs loaded with Schwann cells can yield outcomes comparable to those achieved using autografts.^68,69^ Additionally, neural stem cells, which possess the capacity to differentiate into Schwann cells or secrete growth factors, have been utilized in conjunction with NGCs for neural regeneration.^13,70,71^ To assess the biocompatibility of the 3D-printed CNGs, human neural progenitor cells (hNPCs) were first encapsulated in alginate and then seeded into the 3D-printed CNGs. Following a culture period of seven days, the cells showed typical round morphology in the 3D culture system and remained highly viable within both the PVA and PVA/PEDOT:PSS conduits, suggesting that the 3D-printed CNGs demonstrated a high degree of biocompatibility for cellular growth (Fig. 4E). Our results revealed that the 3D-printed CNGs were extremely biocompatible with NPCs, and their degradation products exhibited no biotoxicity, thereby presenting opportunities for further implantation in animal studies.

To further validate the potential application of the 3D-printed CNGs for neural regeneration, quantitative real-time PCR (RT-qPCR) was conducted on hNPC-containing 3D-printed CNGs. A higher expression level of BDNF was observed in alginate-encapsulated NPCs cultured in the 3D-printed CNGs (Fig. 4E), while there was no significant difference in NTF3 expression between the 2D and 3D culture systems. The 3D culture system has been widely applied in the field of neural engineering since it emulates the native growth and functional characteristics of nervous system cells.^72^ As a member of the neurotrophin family, BDNF plays a key role in mediating the early response of motoneurons to peripheral nerve injury and in promoting subsequent neural regeneration.^73^ It has been reported that enhancing neurotrophic factor secretion leads to better axonal growth.^28,29^ Moreover, BDNF promotes the proliferation of neural stem cells through multiple pathways while facilitating the differentiation of the neuronal precursors.^30,31^ We also noticed a remarkably higher BDNF expression of NPCs cultured in CNGs after electrical stimulation in our previous studies.^13,16^ Consistently, the present qPCR analysis revealed increased secretion of neurotrophic factors by NPCs encapsulated within the 3D-printed CNGs, which may promote both proliferation and differentiation of NPCs, thereby supporting neural regeneration following peripheral nerve injuries.

## 3. Conclusion

In summary, we have developed novel standalone CNGs with customizable dimensions tailored to treat critically sized nerve defects in rodent PNI models. By adjusting the polymer concentration and crosslinking conditions, we fabricated biodegradable and biocompatible 3D-printed CNGs without the support of sacrificial layers. Our future plans include increasing the complexity of CNG design *via* 3D printing and investigating the regenerative potential of 3D-printed CNGs in rodent PNI models for peripheral nerve repair.

## 4. Experimental section

### 4.1. Materials

Polyvinyl alcohol (PVA) (MW ≈ 180 000) and a surfactant-free poly(3,4-ethylenedioxythiophene)–polystyrene sulfonate (PEDOT:PSS) aqueous dispersion (1.1% in H_2_O) were purchased from Sigma-Aldrich. Deionized (DI) water (>18.2 MΩ) was used throughout the study.

### 4.2. Fabrication of crosslinked bioinks

The synthesis of PEDOT:PSS-added aqueous PVA bioinks was modified from existing literature. Briefly, a series of conductive bioinks were prepared with varying weight ratios of PEDOT:PSS and PVA. PVA was first dissolved in DI water to obtain the 16, 20, and 24% PVA dispersions. Subsequently, a constant 10% PEDOT:PSS was introduced into these three PVA dispersions. The mixed bioinks were stirred at approximately 300 rpm for two hours at 90 °C. Bioink gelation occurred following three freeze–thaw cycles consisting of a twelve-hour freeze at −20 °C and a six-hour thaw at room temperature. The crosslinked bioinks were stored at 20 °C until characterization tests were conducted.

### 4.3. Scanning electron microscopy (SEM)

The cross-sectional morphology of the bioinks was observed using a scanning electron microscope (Hitachi S-4800). Bioinks were mixed and prepared according to the method previously described for physical crosslinking. All samples were frozen at −80 °C overnight before lyophilization for 24 hours. Imaging considered the secondary electrons emitted from the sample surface under a beam of primary electrons. The cross-sectional morphology was obtained by cutting the samples to evaluate their internal structures.

### 4.4. Raman spectroscopy

Raman spectra at 532 nm were recorded using a confocal system (Witec, 300AR) to measure the vibrational states and characterize the molecular composition of the bioinks. Each spectrum was obtained through three acquisitions within the range of 0–4000 cm^−1^, and the data were normalized across the different sets.

### 4.5. Conductivity measurement

Electrical conductivity was measured using a digital four-point probe (JG Suzhou Jingge Electronic Co., Ltd) with the corresponding correction coefficient values using the manufacturer's software (v1.2). The diameter and thickness of each sample were measured using a caliper and entered into the four-point probe. Average resistivity measurements were conducted by lowering the four-point probe onto the surface of the bioinks. Resistivity values were measured every ten seconds for a total of ten values per sample. All samples were measured at room temperature. Conductivity was calculated as the inverse of sample resistivity.

### 4.6. Wettability analysis

Wettability was characterized by analyzing the contact angle measured from a single droplet of DI water on the surface of the bioinks. At least three pictures were captured in the horizontal position with a camera. ImageJ software (v1.61) was used to determine the angle of the water droplet with the bioink surface. The contact angle indicates the wettability of the sample surface.

### 4.7. Viscosity and yield stress measurement

Viscoelastic characterizations were conducted based on oscillatory shear rheology using a Discovery Hybrid Rheometer 2 (TA Instruments, New Castle, DE, USA). A sandblasted, 8-mm parallel-plate steel geometry was lowered onto the hydrogel surface until the axial force reached approximately 0.05 N. The stage temperature was set to 25 °C using a Peltier plate temperature controller. Frequency sweeps were measured from 0.1 to 100 rad s^−1^. The average yield stress was derived from the peak stress on the viscosity plots prior to plastic deformation.

### 4.8. Fabrication of 3D-printed conductive nerve guides

The bioinks consisting of PEDOT:PSS and PVA were poured into manufacturer-provided 3D-printing cartridges (Cellink). The bioink cartridges were allowed to physically crosslink using two freeze–thaw cycles consisting of a 24-h freeze at −20 °C followed by a 1-h thaw at room temperature. All bioink cartridges were stored at −20 °C until they were used for 3D printing.

Bioink cartridges were thawed at room temperature for an hour. CNGs were modeled in the shape of a hollow cylinder with a constant 2 mm thickness between the inner and outer diameters matching *in vivo* implantable nerve guides published from our previous work.^13^ The 3D models were rendered using TinkerCAD software. An Inkredible+ Bioprinter (Cellink) was used to 3D print the CAD models using an infill resolution of 0.20 mm and a printing speed of 1 mm s^−1^ on a cold surface. After a successful print, CNGs underwent two freeze–thaw cycles consisting of a four-hour freeze at −20 °C followed by a one-hour thaw at room temperature. CNGs were stored at −20 °C until the compression test was conducted.

### 4.9. Compressive stiffness of 3D-printed conductive nerve guides

Axial compression tests were performed on 3D-printed CNGs. CNGs were placed on a Discovery Hybrid Rheometer 2 (TA Instruments, New Castle, DE, USA) between the stage and a sandblasted 8-mm parallel-plate steel geometry. The stage temperature was set to 25 °C using a Peltier plate temperature controller. The fixture applied a compressive force at 100 µm s^−1^ up to 80% strain or plastic deformation. The stiffness was determined from the linear elastic region of the stress–strain curves up to 15% strain.

### 4.10. Degradability of 3D-printed conductive nerve guides

Degradation tests were performed on 3D-printed CNGs. CNGs were placed in phosphate-buffered saline (pH ≈ 7.4) and soaked overnight at 37 °C before recording the initial weights and pH (*W*_0_). The sample weight was recorded after removing the sample from solution and gently removing moisture with filter paper. The pH of the remaining solution was also recorded using a digital pH meter (sensION MM374) before returning the samples to storage at 37 °C. The weight changes (*W*_1_/*W*_0_ × 100%) reflected the rate of degradation.

### 4.11. Cell culture

Human neural progenitor cells (hNPCs) derived from human induced pluripotent stem cells (iPSCs) were cultured in cell culture plates coated with Matrigel^TM^ Matrix (Corning). Cells were cultured in a complete medium composed of 48% Dulbecco's modified Eagle's medium:nutrient mixture F12 (DMEM/F12, Gibco), 48% neurobasal medium (Gibco), 2% B-27 supplement (Gibco), 1% N-2 supplement (R&D Systems), 1% non-essential amino acids (Cytiva HyClone), 100 mM mL^−1^ 2-mercaptoethanol (BME, Fisher Scientific), 20 ng mL^−1^ recombinant human EGF Protein (EGF, R&D Systems) and 20 ng mL^−1^ recombinant human FGF basic (bFGF, R&D Systems) at 37 °C and 5% CO_2_. The culture medium was replaced every day, and cells were split upon reaching 80% confluency. The first three passages of cells were used for further experiments.

### 4.12. Biocompatibility of 3D-printed conductive nerve guides

Cells were embedded in 1% alginate solution at a density of 1 × 10^6^ cells per mL, and 300 µL of the cell suspension was encapsulated into each nerve guide. The crosslinking solution composed of 99% of the complete medium and 1% calcium chloride (CaCl_2_) was added to all nerve guides and then replaced with the complete medium after 15-min curation. Cells were cultured in an incubator for further experiments, and the culture medium was replaced every day. Live/dead staining was performed to evaluate the cell viability on days 1, 3, and 7, following manufacturer's instructions. Briefly, the culture medium was replaced with a staining solution containing 0.5 µL mL^−1^ of calcein AM (Invitrogen) and 1 µL mL^−1^ of ethidium homodimer-1, and cells were then incubated for 20 min at room temperature. After rinsing with PBS three times, the cross sections of the nerve guides were imaged with a confocal imaging system (Nikon AX R Laser Scanning Confocal). Images were processed using ImageJ (v 1.54f).

### 4.13. Quantitative real-time polymerase chain reaction (RT-qPCR)

NPCs were cultured in 6-well plates and in the 3D-printed CNGs composed of 20% PVA/PEDOT:PSS encapsulated in alginate. RT-qPCR was conducted according to the manufacturer's instructions. The iScript cDNA Synthesis Kit (Bio-Rad) was used for RNA reverse transcription. A QuantStudio™ 5 Real-Time PCR System was used for conducting RT-qPCR. The RT-qPCR reaction mixture was prepared using TaqMan Gene Expression Master Mix and TaqMan primers (Thermo Fisher Scientific) glyceraldehyde-3-phosphate dehydrogenase (GAPDH Hs02786624_g1), neurotrophin 3 (NTF3 Hs00267375_s1) and brain derived neurotrophic factor (BDNF Hs02718934_s1). The analysis was performed using the ΔΔCT method, and the gene expression levels were normalized by GAPDH.

### 4.14. Statistical analysis

At least three samples were tested for experiments in each group. Independent Student's *t*-tests were performed for comparison between two groups. One-way or two-way analysis of variance (ANOVA) was used for the comparison of differences among multiple groups followed by Tukey's multiple comparisons test for evaluating differences between groups. Data were analyzed using GraphPad Prism software (v10.0.2) and a *p*-value < 0.05 was considered statistically significant. All data are presented as mean ± standard error of mean.

## Author contributions

S. S. conceptualized this study, acquired funding, supervised the project, and designed the overall methodology. A. H., R. G., and Y. T. K. fabricated the bioinks, printed the conductive nerve guides, and validated the 3D-printing and freeze–thaw processes. L. L. performed the characterization of the crosslinked bioink. A. H., R. G., and Y. T. K. conducted the physical and mechanical property measurements. L. L. performed the cell live/dead staining and RT-qPCR analysis. S. S., A. H., R. G., and Y. T. K. analyzed and visualized the data. A. H., R. G., and Y. T. K. wrote the initial draft of the manuscript. S. S. and L. L. reviewed and edited the manuscript. The manuscript was written through the contributions of all authors. All authors have given approval to the final version of the manuscript.

## Conflicts of interest

All authors declare no conflicts of interest.

## Supplementary Material

MA-007-D5MA00932D-s001

## Data Availability

All data generated or examined during this research are provided within this article. Further data can be accessed upon reasonable request. Supplementary information: images of conductive bioinks; 3D-printed conductive nerve guides with varying experimental conditions. See DOI: https://doi.org/10.1039/d5ma00932d.
