# Supplementary material for: Engineering 3D-printed standalone conductive nerve guides using soft bioinks for peripheral nerve injuries
Source: Mater Adv. 2025 Nov 12;7(1):606–17. doi: 10.1039/d5ma00932d (PMC12666618; doi:10.1039/d5ma00932d)
Supplement: MA-007-D5MA00932D-s001 [file MA-007-D5MA00932D-s001.pdf]

Supplementary Information (SI) for Materials Advances.
This journal is © The Royal Society of Chemistry 2025

Supporting Information

Engineering 3D-printed standalone conductive nerve guides using soft bioinks for peripheral nerve injuries

Lin Li†, Angel Hernandez†, ‡, Ryan Grevsmuehl†, ‡, Yu-Ting Kou†, Shang Song†, ††, *

† Department of Biomedical Engineering, College of Engineering, University of Arizona,
†† Departments of Materials Science and Engineering, Neuroscience GIDP, and BIO5 Institute, University of Arizona, Tucson, Arizona, 85721, US
‡ These authors contributed equally to the manuscript
* Corresponding author; shangsong@arizona.edu

Tables

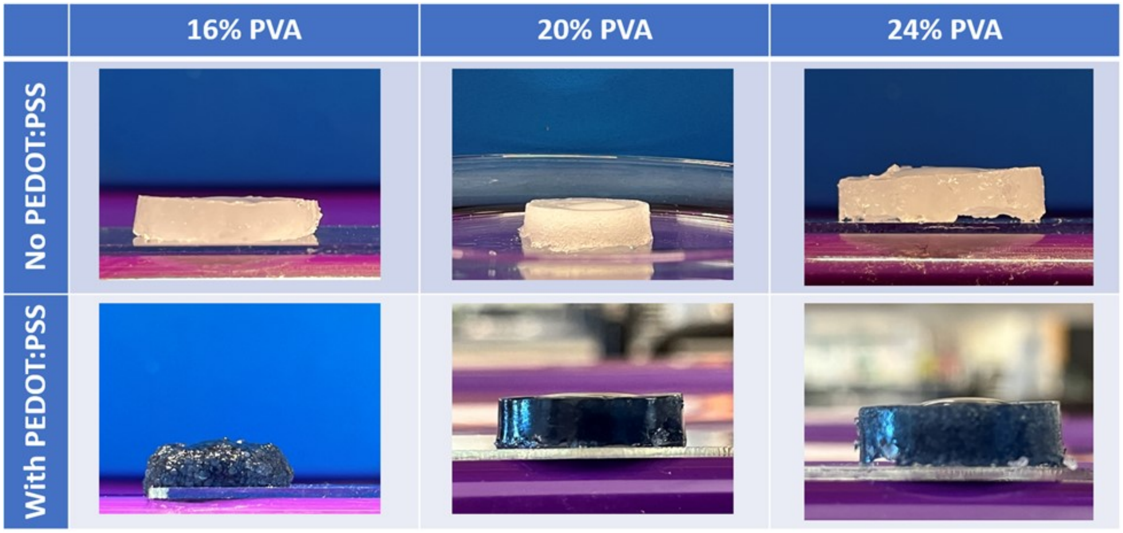

Table S1. Crosslinked conductive bioinks. Bioinks underwent gelation using a freeze-thaw cycle consisting of a twelve-hour freeze at −20°C and a six-hour thaw at room temperature. The presence of PEDOT: PSS resulted in dark color while no gross change in morphology was detected for PVA at various concentrations.

	Immediate after making solution (t freeze = 0, thaw time = 0)	1 hour freeze 1 hour thaw 2x	After freezing for 12 hours, 1 hour thaw 2x	After freezing for 24 hours, 1 hour thaw 2x
Dimensions:<br>4 mm in<br>6 mm out<br>5 mm height				

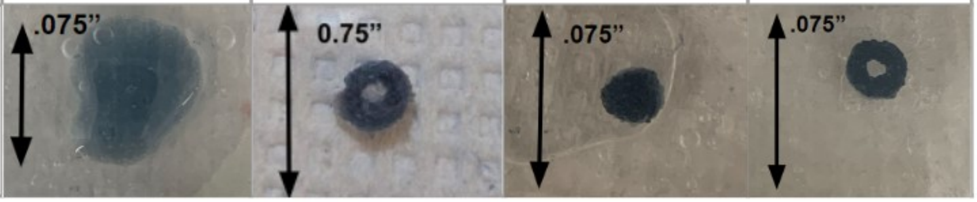

Table S2. The effect of freeze-thaw process on 3D-printed CNGs. A visual comparison highlighted the structural differences of bioinks at varying freeze-thawing periods. All bioinks consisting of 20% PVA with PEDOT: PSS experienced a fixed one-hour thawing time with variable freeze time from immediately after the solution was made (left: control) up to 24 hours of freezing (right).
